# Nomogram of age-specific anti-Müllerian hormone levels in healthy Egyptian females

**DOI:** 10.1371/journal.pone.0254858

**Published:** 2021-07-26

**Authors:** Eman Ahmed El-Attar, Tamer A. Hosny, Kiyoshi Ichihara, Rania N. Bedair, Ahmed Salah El-Din Tork

**Affiliations:** 1 Chemical Pathology Department, Medical Research Institute, Alexandria University, Alexandria, Egypt; 2 Department of Obstetrics and Gynecology, Faculty of Medicine, Alexandria University, Alexandria, Egypt; 3 Faculty of Health Sciences, Department of Clinical Laboratory Sciences, Yamaguchi University Graduate School of Medicine, Ube, Japan; Universita degli Studi dell’Insubria, ITALY

## Abstract

**Background:**

Anti-Müllerian hormone (AMH) is an important determinant of ovarian reserve in fertility workups in many clinical settings. Thus, we investigated the age dependent decline in AMH specific to the Egyptian population and sought to establish an age dependent reference interval parametrically.

**Methods:**

Serum samples were collected from 841 apparently healthy women. AMH was measured using an electro-chemiluminescent technique. Box-Cox power transformation was used to make the AMH distribution Gaussian for parametric derivation of reference intervals.

**Results:**

Power of 0.4 was found optimal for Gaussian transformation of AMH reference values. We demonstrate the strong negative relation between circulating AMH and female age with Spearman’s correlation coefficient of r_S_ = −0.528. Age-specific reference interval was determined for every 5 years of age from 16 to 49, and nomogram was constructed by smoothing the lines connecting adjacent lower and upper reference limits.

**Conclusion:**

The age-specific reference intervals and the age-AMH nomogram could be valuable in the clinical practice of in reproductive medicine. To our knowledge, this is the first study to confirm AMH levels in Egyptian females. We were able to explore age-related AMH levels specific to Egyptian females in the fertile age group and to treat skewed AMH data in a multi-step scheme using power transformation. Thus, a more accurate nomogram was constructed accommodating a profile delineated for a wide age range and a rescaled AMH axis improving its usability.

## Introduction

The concept of ovarian reserve which reflects the quantity and quality of oocytes is an essential topic as assisted reproduction is gaining popularity over the years. Among markers of ovarian reserve, AMH has gained particular interest as it exhibits very low intra and inter cycle variability. AMH is clearly related to ovarian function as it is secreted by the granulosa cells of developing follicles until they reach a suitable size and differentiation state under the influence of pituitary FSH [1, 2]. Hence, serum AMH levels essentially reflect the ovarian follicular pool [3]. AMH was also found to be superior to antral follicular count and FSH levels in predicting menopause onset [4].

AMH is secreted by the ovary into circulation, hence AMH is measurable in serum. Interestingly, in women circulating AMH appears to be exclusively of ovarian origin. Following bilateral ovariectomy, AMH levels reach undetectable values within 3–5 days [5]. In clinical settings, the low inter- and intra-cycle variability in serum AMH levels permits random timing of AMH measurement during the menstrual cycle. In women, AMH levels seem to be unmodified under conditions in which endogenous gonadotrophin release is substantially diminished, such as during pregnancy, GnRH agonist and short-term oral contraceptive treatment. This indicates that non-cyclic, FSH-independent ovarian activity persists even when pituitary FSH secretion is suppressed [6].

Previous studies have reported important clinical features and utilities of serum AMH including decline with aging, a good correlation with oocyte yield in assisted reproduction, increased levels in polycystic ovarian syndrome, and decreased levels following ovarian surgery and toxic treatment. Recent studies, including meta-analysis studies and clinical trials together with the new generation AMH assays that enabled accurate measurement of very low serum concentrations, have yielded firm clinical interpretations of serum AMH in the past few years. [7–9]

Age-independent standardization of AMH values was suggested to compare ovarian reserves among women at different ages [7]. Validation of an age-related AMH nomogram will enable better clinical counseling concerning reproductive capacity [10]. We aim to define the decline in circulating AMH with age and develop and validate an age-related nomogram in Egyptian women.

Despite the strong association between AMH levels and age, age-adjusted reference intervals have not been introduced to clinical practice. This could be a new approach to clinical judgement of fertility & ovarian reserve in different age groups. To our knowledge, this is the first study to explore age-related AMH levels in Egyptian females.

## Methodology

### Patient cohort

From January 2018 to March 2019, 998 apparently healthy females attending the Alexandria University hospital laboratory for AMH analysis were recruited in this study. Written informed consents were obtained from all participating females. The Ethical Committee of Medical Research Institute, Alexandria University approved this work. All study procedures were performed in accordance with the ethical standards of the 1964 Helsinki declaration and its later amendments.

**Fig 1:**
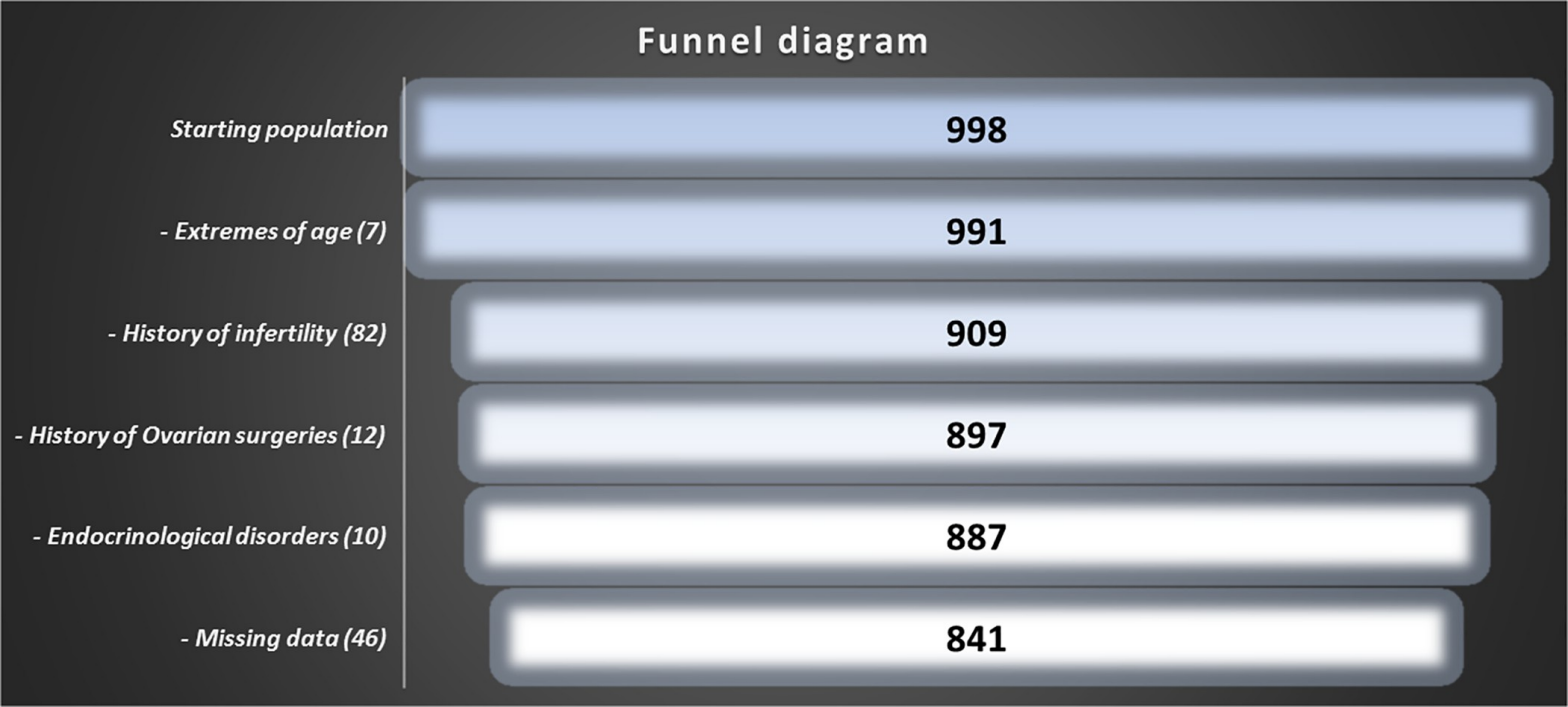
Data selection procedures and sample sizes.

All participating females were asked to fill out a questionnaire form that included questions about age, childbearing, previous surgeries, history of infertility, and history of polycystic ovary syndrome (PCOS). Women who had conceived and bore children were considered to have proven fertility. We excluded females having medical history or other laboratory tests suggestive of infertility or PCOS, previous ovarian surgeries, unexplained infertility or those having any endocrinological disorders. Participants were studied cases by case to exclude any unclear or missing data. Women who had visited infertility clinics and were informed of no pathology were included in the study. Cases of infertility were included only if the reason was male infertility. The study started with 998 females and funneled down to 841 females after excluding the above conditions. Thus, this study population represents a normal fertile Egyptian female population (**Fig 1**).

### AMH assay

AMH assay was performed using electrochemiluminescence technology on cobas e 601 module, Roche using Elecsys AMH kit. Results were expressed in ng/mL. Intraassay and interassay coefficient of variation were 1.7% & 3.5%, respectively.

### Statistical analysis

Data was collected, tabulated and patients with missing age or AMH values were excluded. The parametric method was used to calculate the AMH RI Since the distribution of our AMH test results was non-Gaussian, with a great degree of positive skewness, we used power transformation based on the Box-Cox formula [11]

*X* = (*x^p^*−1)/*p* …………………when p ≠0.0

*X* = log(*x*) ………..…………….. when p = 0.0

formula 1: (The Box-Cox formula)

where x and X denote observed value before and after transformation, and the ‘p’ represents the power of the transformation.

**Fig 2:**
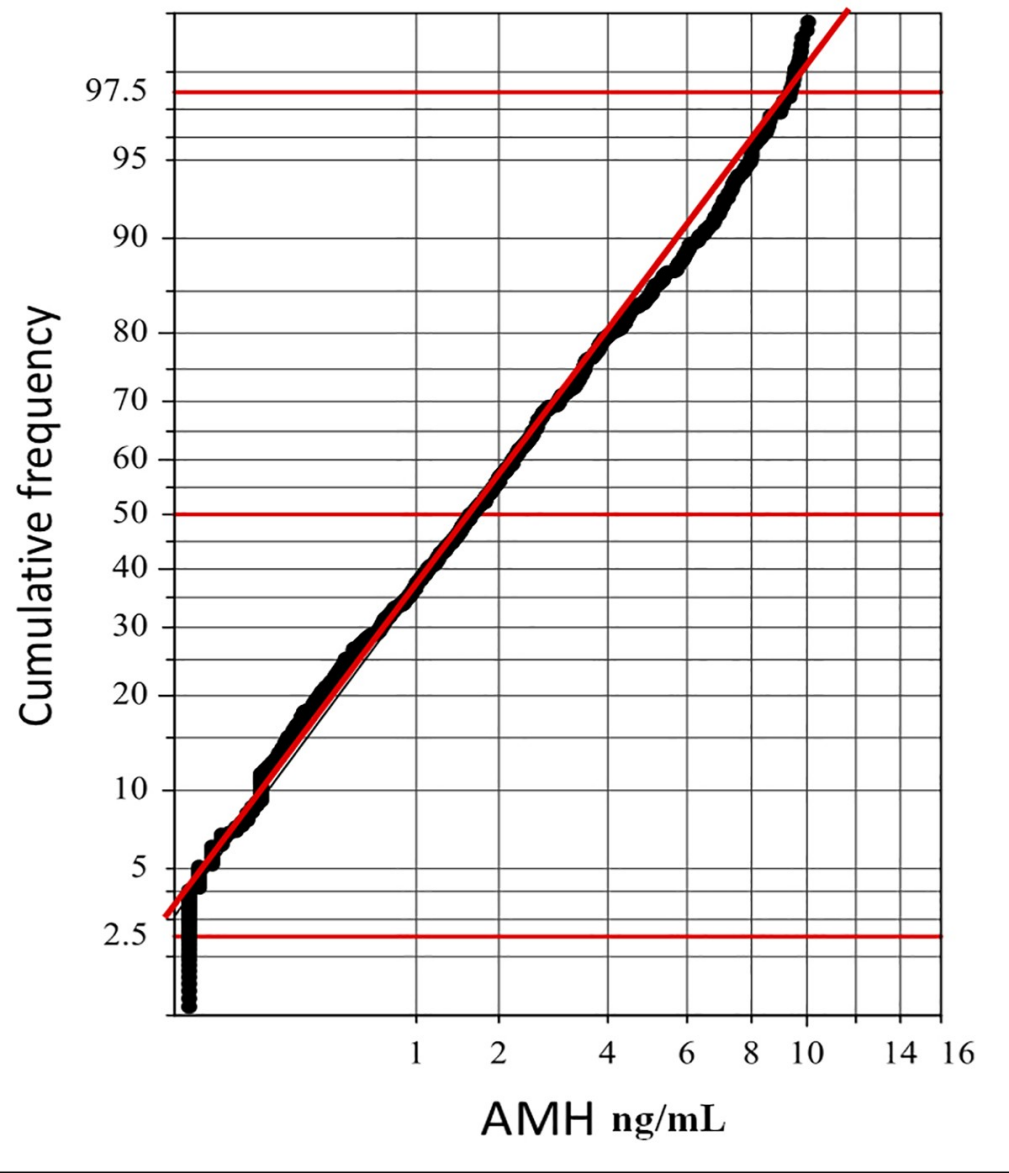
Confirmation of Gaussian transformation of AMH values by probability plot. The vertical axis represents the cumulative frequency (P %), transformed as log [(100 − P)/P]. The horizontal axis represents AMH test results. This axis was transformed by use of Box–Cox formula using p = 0.4 as an optimal power. The linearity of the segment between 10 and 90% indicates successful Gaussian transformation by use of the formula.

The ‘p’ value was empirically determined by use of probability plot. The ‘p’ was changed stepwise from 1.0 to 0.0 with decrement of 0.1, and an optimal ‘p’ value (p_O_) was determined as 0.4 that gave the longest linear segment in the probability plot **(Fig 2)**

To make a nomogram for age-specific reference intervals, the following procedure was used:

1. AMH dataset were power transformed using p_O_ to approximate a Gaussian distribution.
2. A paired dataset with data size N composed of age (Xi) and test result (Yi) [i = 1 to N] were multiplied by R times (R = 10) to inflate the size to N×R. In this process of multiplication, a flat random value between −2.0 to 2.0 was added to Xi (age), and that between −0.2 and 0.2 were added to Yi (AMH^0.4^),
3. The dataset was then stratified by an age block of every 5 years, and block-wise summary statistics of mean (M_k_^T^) and SD (SD_k_^T^) were calculated. Then, the 95% confidence interval for the block (k) was derived as M_k_^T^ ± 1.96×SD_k_^T^ (or LL^T^ = M_k_^T^ −1. 96×SD_k_^T^; UL^T^ = M_k_^T^ + 1.96×SD_k_^T^), where LL and UL represent lower limit and upper limit of the RI, respectively.
4. Then, LL^T^ and UL^T^ were reverse transformed to obtain the LL and UL at the original scale.
5. The vertical data block by age was moved upwards by one year of age for calculating the summary statistics in a stepwise fashion.
6. Curves connecting the summary statistics were smoothed using the Bezier curve function of Microsoft Power Point.

## Results

**Fig 3:**
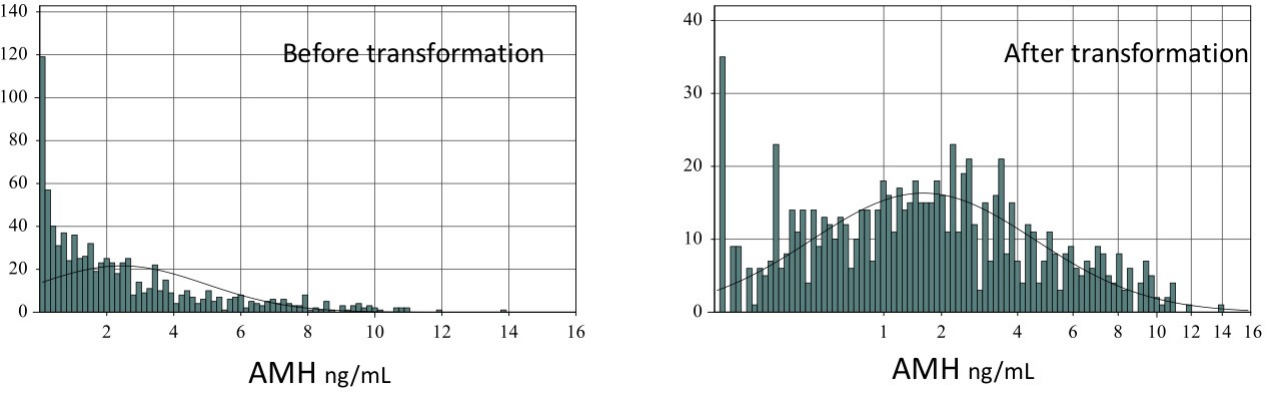
Data distribution of AMH before (left) and after transformation where the scale is adjusted to fit the AMH power transformation (right).

**Fig 4:**
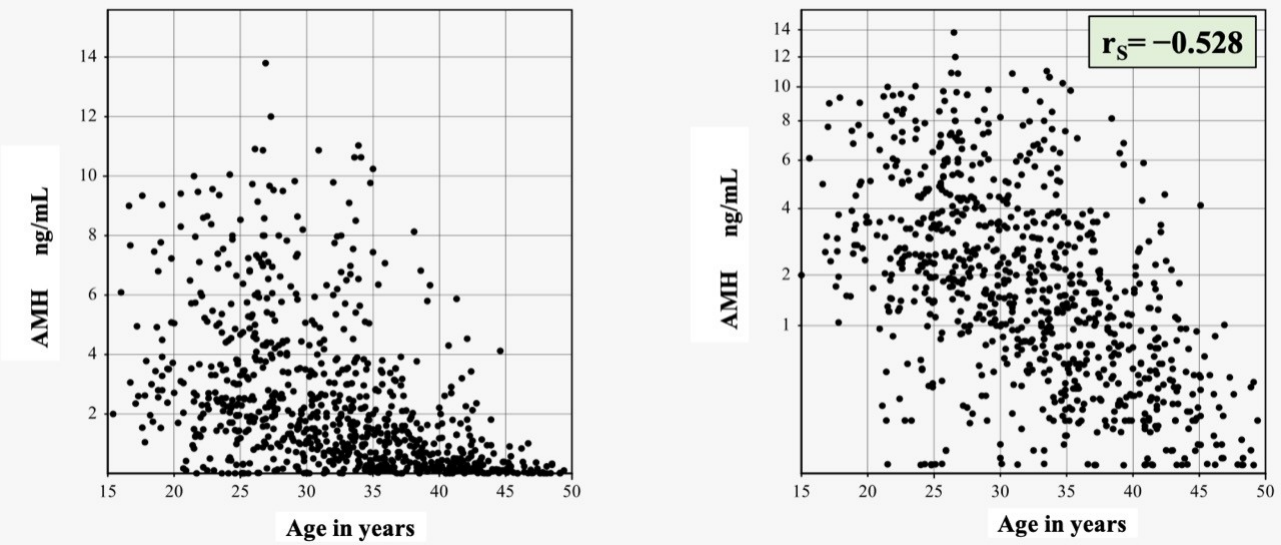
Scatterplot of AMH versus age without (left) / with adjustment of the scale of AMH axis to fit the AMH power transformation (right).

The distributions of AMH among 841 apparently healthy Egyptian females are shown in **Fig 3** without (left) and with transformation by setting p = 0.4 (right). The relationship of AMH and age was drawn, as shown **Fig 4**, without (left) and with power transformation of the Y-axis to fit the transformation (right). The degree of association between age and AMH calculated as Spearman’s correlation (rS) was −0.528.

**Fig 5:**
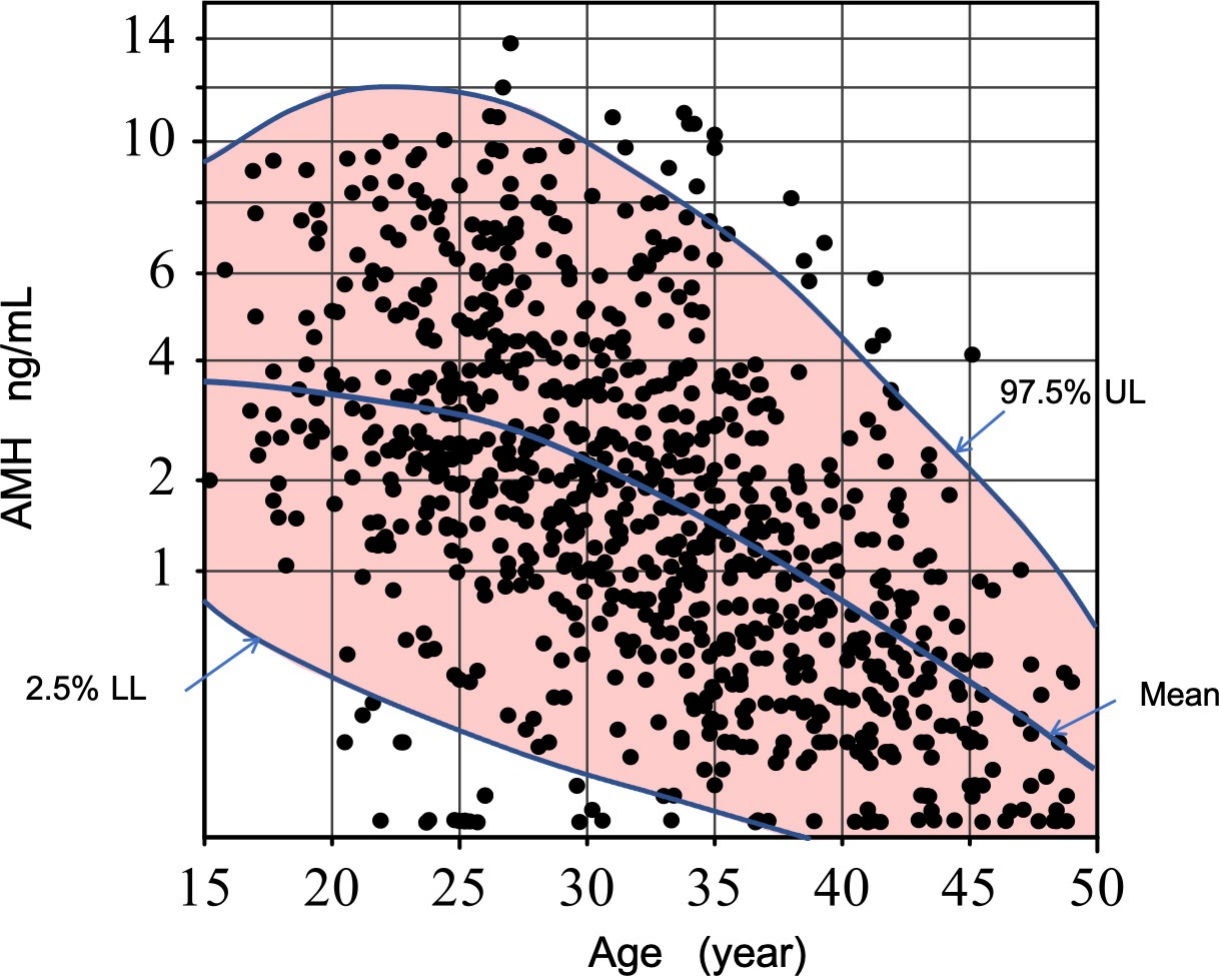
Nomogram of age-related profile of Antimullerian hormone (AMH). The scale is adjusted to fit the AMH power transformation. The central lines and the outer margins of the colour shades represent the median and the fifth and 95th centiles for the year-by-year moving vertical 10-year segment.

The nomogram of age-related profile of median and 95% CI (LL and UL) of AMH was produced as shown in **Fig 5** by the scheme described in the Methods. The age specific RIs that were determined in every 5 years of age from 16 to 49 are shown in **Table 1.** The scale of the AMH axis in our nomogram was also adjusted to fit our AMH power transformation, Thus, AMH reference interval for any age can be directly deduced from our nomogram (Fig 5).

**Table 1:** Reference intervals of AMH determined for every 5 years of age from 16 to 49.

			Reference interval for AMH (ng/mL)		
Age	Mid-Age	n	LL	Me	UL
15–19	17	27	0.51	3.53	10.4
20–24	22	113	0.08	3.00	12.4
25–29	27	192	0.08	2.80	11.6
30–34	32	195	0.02	1.88	8.6
35–39	37	164	0.00	1.13	6.4
40–44	42	109	0.00	0.51	3.4
45–49	47	42	0.00	0.14	1.4

LL = lower limit; Me = median; UL = upper limit.

## Discussion

Serum AMH level is a marker of continuous non-cyclic growth of ovarian follicles. To date, the ovarian reserve of primordial follicles cannot be clinically assessed. We describe the relationship between AMH and age of Egyptian women in the fertile age group (**Figs 2 & 3**). We used a parametric transformation (Fig 3). Our results point to a strong negative correlation between circulating AMH and age where r_S_ = − 0.528. The highest average AMH was in the group of women aged less than 20 years (**Fig 4**). This relationship has been tackled in several studies around the world [8]. A study was conducted in 2011 on a large scale of 9,601 females to describe the decline in AMH levels with age in the United Kingdom. The study was conducted on 4,590 women, then validated in 4,588 women and finally validated on 423 women known to be ovulating, aged 25–45 years. They developed a nomogram based on AMH and age and used 5 regression models: linear, biphasic, differential, power and quadratic. For each model, the mean, standard deviation (SD), median, quartiles, and range of residuals are reported, as well as the sum of absolute and squared residuals and R^2^ statistic (proportion of variance explained by the model). R^2^ values ranged from 19.45% to 19.48% in the training dataset and from 21.30% to 21.36% in the validation dataset, suggesting very little difference in goodness of fit. They concluded that four nonlinear regression models (esp power and quadratic model) provide the best fit. Although their work used a much larger sample size, our study cover a larger age range in the reproductive years of females (15–49 years), yet we found a stronger relationship with age. Also, our fully automated AMH assay method shows a superior performance with excellent precision and sensitivity: i.e., intraassay and interassay CVs of 1.7% and 3.5%, respectively, at mid-normal level, and the limit of quantitation of 0.01 pmol/L. Whereas intra-assay CVs at the low, mid, and high concentration levels of the method used in the UK study were 3.4%, 6.3%, and 8.6%, and interassay CVs were 11.4%, 8.4%, and 15.4%, and the lower limit of detection was 0.2 pmol/L. Kelsey et al also described a validated model of AMH decline from conception to menopause. Their model was generated from a study of 3,620 healthy subjects from preterm infants to 54.3 years of age. Their model has an R2 of 0.34 with coverage of much wider age-range [12].

De Vet et al found that AMH peaks around the age of 20 years then declines [13]. The finding of the report agrees with our data of the highest average AMH in the group of women aged less than 20 years. While other studies report that serum AMH peaks at the age of 25 [14]. A recent study shows that AMH measured by Roche Elecsys highly correlated with antral follicle count and retrieved oocyte count after ovarian stimulation. Roche Elecsys AMH assay is traceable to the Beckman Coulter AMH Gen II ELISA (unmodified version without predilution) assay [15]. The different assay principle and reagents used in the measurement should account for a considerable part of the conflict between results of different studies over the years. Thus, the call for harmonization of AMH assays is on the rise which should reduce bias and facilitate the comparison of results [16].

The utility of AMH testing in assisted reproduction is widely discussed in the recent years. Firm evidence points to a linear correlation between serum AMH and oocyte yield. Serum AMH was suggested as the best marker to predict poor or hyper responders. Thus, integration of serum AMH in nomograms to optimize the ovarian stimulation protocol was suggested in order to reduce the risk of cycle cancelation or ovarian hyperstimulation [17].

Given that our source of data were laboratory results, the only possible source of selection bias in this work may be that the females participating in this study were seeking medical assistance and laboratory service, hence they may not be healthy women. Yet we largely overcame this issue through exclusion of cases using the questionnaire where medical history or other laboratory tests show signs of infertility or polycystic ovary syndrome. Participants were studied cases by case to exclude women who had missing data or unclear responses to the questionnaire. Cases of infertility were included only if the reason was male infertility. In PCOS, there is an increase in the number of preantral follicles [18], Furthermore, AMH production is increased in anovulatory PCOS women by granulosa cells [19], resulting in increased plasma AMH levels in PCOS [20, 21]. Frank symptomatic polycystic syndrome was deliberately excluded in this study. However, asymptomatic ovulating polycystic ovary cases were not excluded as they exist in a small fraction of the fertile Egyptian female population.

Two recent studies suggest inter and intracycle variations during ovarian stimulation might be explained by factors other than analytical variability. AMH levels were higher in the follicular phase than the luteal phase [22]. The average intraindividual AMH variability in a menstrual cycle was 20% and this biological variation was at least twice the analytical variation (Hadlow et al., 2016) [23]. Short-term inter-cycle variation might be due to the biological variation in the number of AMH-producing follicles per cycle [24].

We demonstrate in a large cohort of Egyptian females that the circulating AMH concentrations exhibit a decline with age that is well modeled. Further validation in a similar population with clinically proven fertility and phenotyping will provide further confirmation.

To our knowledge, this is the first study to confirm age-related AMH reference intervals specific to Egyptian females in the fertile age group. Our results emphasized the strong negative correlation of AMH with age in fertile Egyptian females, introducing this nomogram in clinical practice would guarantee better assessment of fertility and ovarian reserve in different age groups of Egyptian females with their specific genetic, nutritional, and environmental profiles.

Compared to previous studies, fully automated AMH assay method shows a superior performance whether in precision or sensitivity. Our new scheme of determining the reference intervals and delineation of age-related profile (nomogram) using the power transformation of AMH values is considered a much-improved approach. In previous studies, the nomograms were constructed by non-linear regression analysis of the observed age-AMH curve. Although appropriate fitting of the curve to the entire age range was difficult, our new scheme worked well to delineate the profile for the required wide age range.

Finally, adjusting the scale of the AMH axis in our nomogram to fit our AMH transformation, would make it possible to directly deduce the AMH reference interval for any age without the need for any post-calculations, which would certainly improve its usability.

## Supporting information

S1 File (XLSX)
